# The Use of Activated Micronized Zeolite Clinoptilolite as a Possible Alternative to Antibiotics and Chestnut Extract for the Control of Undifferentiated Calf Diarrhea: An In Vitro and In Vivo Study

**DOI:** 10.3390/ani10122284

**Published:** 2020-12-03

**Authors:** Constantin Cerbu, Vlad Alexandru Ilaș, Michał Czopowicz, Adrian Valentin Potârniche, Elisa-Paz Bodart-Nieva, Elena Andruța Mureșan, Jarosław Kaba, Marina Spinu, Emoke Pall

**Affiliations:** 1 Department of Infectious Diseases, Faculty of Veterinary Medicine, University of Agricultural Sciences and Veterinary Medicine Cluj-Napoca, Calea Manastur 3-5, 400372 Cluj-Napoca, Romania; adrian.potarniche@usamvcluj.ro (A.V.P.); elisapazbodartnieva@gmail.com (E.-P.B.-N.); marina.spinu@usamvcluj.ro (M.S.); emoke.pall@usamvcluj.ro (E.P.); 2 Department of Reproduction, Faculty of Veterinary Medicine, University of Agricultural Sciences and Veterinary Medicine Cluj-Napoca, Calea Manastur 3-5, 400372 Cluj-Napoca, Romania; vlad.ilas@usamvcluj.ro; 3 Division of Veterinary Epidemiology and Economics, Institute of Veterinary Medicine, Warsaw University of Life Sciences-SGGW, Nowoursynowska 159c, 02-776 Warsaw, Poland; michal_czopowicz@sggw.edu.pl (M.C.); jaroslaw_kaba@sggw.pl (J.K.); 4 Food Engineering Department, Faculty of Food Science and Technology, University of Agricultural Sciences and Veterinary Medicine Cluj-Napoca, Calea Manastur 3-5, 400372 Cluj-Napoca, Romania; andruta.muresan@usamvcluj.ro

**Keywords:** micronized zeolite, clinoptilolite, calves, diarrhea

## Abstract

**Simple Summary:**

Today, zeolite has appeared as an interesting alternative for the symptomatic treatment of acute diarrhea. Therefore, this study aimed to investigate the properties of activated micronized (five microns) zeolite clinoptilolite (MZC) from Transylvania, Romania, first by testing it in vitro and then in vivo on calves with diarrhea. To assess the toxic potential of MZC, we performed a cell cytotoxicity assay on cells of bovine origin, while its antimicrobial activity was investigated on *Escherichia coli*. The uncontrolled in vivo study was carried out over 8 days on a fattening farm, with some 650 calves. Selected calves were randomly assigned to 4 groups of 20 individuals. Several combinations between activated MZC, chestnut extract, and oxytetracycline were tested. At the beginning of the study, all calves had diarrhea, while at the end of the study, the prevalence of diarrhea was significantly lower in all four groups (*p* < 0.001), including the ones treated with MZC. Due to its low cytotoxicity on the intestinal cells and with regards to the results we obtained in vivo, MZC could represent an alternative method to reducing the amount of antimicrobials needed for the symptomatic treatment of diarrhea in calves, therefore contributing to the reduction of the antimicrobial resistance phenomenon.

**Abstract:**

Today, zeolite appears as an interesting alternative for the symptomatic treatment of acute diarrhea. Therefore, this study aimed to investigate the properties of activated micronized (5 microns) zeolite clinoptilolite (MZC) from Transylvania, Romania, first by testing it in vitro and then in vivo on calves with diarrhea. To assess the toxic potential of the MZC, we performed a cell cytotoxicity assay using the MTT (3-(4,5-dimethylthiazol-2-yl)-2,5-diphenyltetrazolium bromide) technique on primary bovine intestinal epithelial cells (BIECs). The antimicrobial activity of MZC was investigated by measuring the minimal inhibitory concentration (MIC) on *Escherichia coli* (ATCC 25922). The uncontrolled in vivo study was carried out over 8 days on a fattening farm, with some 650 calves. Selected calves were randomly assigned to four groups of 20 individuals. Several combinations between MZC, chestnut extract, and oxytetracycline were tested. At the beginning of the study, all calves had diarrhea, while at the end of the study, the prevalence of diarrhea was significantly lower in all four groups (*p* < 0.001), including the ones treated with MZC. Due to its low cytotoxicity on the intestinal cells and with regards to the results we obtained in vivo, MZC may be considered an alternative for the symptomatic treatment of undifferentiated diarrhea in calves.

## 1. Introduction

Zeolites possess distinctive and outstanding physicochemical characteristics. These properties make them useful in a variety of applications, including agronomy, ecology, certain manufacturing, industrial processes, medicine, and cosmetics [1]. The diversity of zeolites’ application is a consequence of their unique porous structure. Thus, pores form negatively charged channels and cavities occupied by positively charged alkali; alkali earth monovalent (i.e., Na+, K+); and divalent (i.e., Ca^2+^) ions, OH-groups, or H_2_O molecules can effortlessly exchange these with other molecules and cations from the proximity. Lately, the application of a specific natural zeolite material, clinoptilolite, has been documented in human and veterinary medicine. Many recognized positive medical effects of clinoptilolite have been attributed to its basic material properties, particularly to its reversible ion-exchange and adsorption capacity [1,2,3,4]. This important feature of clinoptilolite, potentially related to the removal of toxic agents, may be seen as a support to homeostasis, therefore offering multiple benefits and potential use in different pathologies [1]. As a consequence, clinoptilolite use in animal biotechnology and veterinary medicine, because of its detoxifying, antioxidant, hemostatic, growth-promoting, and immunostimulating effects, is soundly supported [5].

Cattle producers consider calf diarrhea a significant cause of economic loss, as it is responsible for more than half of registered calf mortality on dairy farms [6]. Multiple microorganisms play a key role in this pathology, such as bovine rotavirus (BRV), bovine coronavirus (BCoV), bovine viral diarrhea virus (BVDV), *Salmonella enterica*, *Escherichia* (*E.*) *coli*, *Clostridium perfringens*, and *Cryptosporidium parvum*, along with newly emerging enteric viruses such as bovine torovirus (BToV) and caliciviruses (bovine norovirus (BNoV) and Nebovirus) [6,7]. The classical treatment of diarrhea in calves includes parenteral administration of antibiotics with a predominantly Gram-negative spectrum of activity, parenteral administration of non-steroidal anti-inflammatory drugs [8], and intravenous (iv) fluid therapy that is primarily based on the degree of dehydration. A decrease of more than 8% body weight is believed to require intravenous (iv) fluids rather than exclusive oral fluids for successful rehydration [9]. In addition to classical therapy, alternatives such as the homeopathic treatment [10] or the use of chestnut extract have been proposed [11].

Since diarrhea remains one of the most prevalent health challenges in both the beef and dairy industries, the use of clinoptilolite with anti-diarrheic properties seems to be well-motivated.

The abusive and occasionally uncontrolled use of antimicrobials in veterinary practice accelerates the continuously increasing number of resistant bacteria strains [12]. Thus, research that may ultimately lead to more specific treatment and control recommendations for calf diarrhea is strongly encouraged. Research on possible applications of clays and zeolites in the biomedical sector is steadily increasing, and, in the context of antibiotic resistance, mineral-based therapies against bacterial infections have become a subject of interest [13]. As described in several in vitro and in vivo studies, the properties of natural zeolites suggest that zeolite may be clinically beneficial when given orally in patients with digestive disorders. Preliminary results on the medical use of zeolite obtained by Grce and Pavelić (2005) [14] indicate antiviral properties of clinoptilolite that opens possibilities for therapeutic use. Moreover, clinoptilolite has also been used as an anti-diarrheic drug in humans [15]. In this framework, the aim of the paper was to investigate the properties of activated micronized zeolite clinoptilolite (MZC) from Transylvania, Romania, primarily by testing its in vitro and further in vivo effects on enterocytes and on calves with diarrhea, respectively. Therefore, during the experiment, we aimed to (1) assess the safety of micronized clinoptilolite by testing its cytotoxicity, (2) evaluate the potential of MZC as a symptomatic treatment in calves with acute diarrhea, (3) evaluate its capacity to replace the antibiotics and chestnut extract used for the symptomatic treatment of diarrhea in calves, and (4) compare its efficacy with routine farm therapy against calf diarrhea.

As far as we know, this is the first study in which the properties of activated MZC has been investigated in calves.

## 2. Materials and Methods

### 2.1. Activated Micronized Zeolite Clinoptilolite: Source and Composition

The micronized zeolite clinoptilolite (MZC), with an average diameter of the particles of 5 microns, was purchased from Zeolites Production S.A (Cluj-Napoca, Romania), from Rupea Zeolite Mines located in the central region of Romania. The chemical composition of the MZC used in this study is presented in Table 1. Prior to its use, the MZC was activated in a rotary oven at 300 °C for 40 min.

### 2.2. Cytotoxicity Assessment

#### 2.2.1. Cell Cultures of Primary Bovine Intestinal Epithelial Cells (BIECs)

Calf jejunal tissue was harvested from a slaughterhouse in sterile conditions and immersed in phosphate-buffered saline (PBS) solution supplemented with 1% antibiotic-antimycotic (Gibco, Thermo Fisher Scientific, Waltham, MA, USA). The sample was washed in ice-cold PBS (Sigma-Aldrich, St. Louis, MO, USA), and the mucosa was minced (2 cm^2^) using a surgical scalpel, then treated with collagenase I (Sigma-Aldrich, St. Louis, MO, USA) and 0.1 mg/mL Dispase (Sigma-Aldrich, St. Louis, MO, USA) for 45 min. The resulting suspensions were filtered (74 µm nylon mesh) and centrifuged at 200× *g* at 4 °C for 7 min. The cell pellet was re-suspended in Dulbecco’s Modified Eagle Medium (DMEM)/F12 (Sigma-Aldrich, St. Louis, MO, USA) supplemented with 10% fetal bovine serum (Sigma-Aldrich, St. Louis, MO, USA), 1% antibiotic-antimycotic (Gibco, Thermo Fisher Scientific, Waltham, MA, USA), 1% glutamine (Sigma-Aldrich, St. Louis, MO, USA), 1% non-essential amino acids (NEA, Sigma-Aldrich, St. Louis, MO, USA), 15 ng/mL EGF (epidermal growth factor; Sigma-Aldrich, St. Louis, MO, USA), and 1 µg/mL hydrocortisone (Lonza, Basel, Switzerland), and incubated at 37 °C with 5% CO_2_. After 72 h, the medium was changed, and 7 days later, the first passages were performed.

#### 2.2.2. MTT Assay

To assess the toxic potential of the MZC, we performed a cell cytotoxicity assay using the MTT (3-(4,5-dimethylthiazol-2-yl)-2,5-diphenyltetrazolium bromide) technique on BIECs, prepared as previously indicated. The cells were seeded at a density of 10^5^ cells per well in 96-well flat-bottom plates and allowed to adapt for 24 h under physiological growth conditions. The cultures were then exposed for 24 h to MZC at increasing concentrations, ranging from 19.5 μg/mL to 10 mg/mL. Cell cultures treated only with the medium were used as controls. After 24 h, the cells treated with zeolite were incubated for 2 h with 150 μL of MTT. The MTT assay involves the conversion of the water-soluble MTT (3-(4,5-dimethylthiazol-2-yl)-2,5-diphenyltetrazolium bromide) to insoluble formazan. The formazan was solubilized with 100 μL DMSO, and the absorbance of each sample was read at 490 nm using an ELISA microplate reader (Bio-Rad, Hercules, CA, USA). Cell viability (%) was calculated by using the equation: cell viability (%) = OD test × 100/OD control. The experiment was carried out in triplicate.

### 2.3. Antimicrobial Activity

The antimicrobial activity of MZC was investigated by measuring the minimal inhibitory concentration (MIC) on ATCC 25922 *E. coli* strain. For that, the Mueller–Hinton broth dilution technique was used, as recommended by the Clinical and Laboratory Standards Institute (CLSI) [16]. Serial dilutions of activated MZC (10 mg/mL, 5mg/mL, 2.5 mg/mL, 1.25 mg/mL, 625 µg/mL, 312.5 µg/mL, 156 µg/mL) were prepared in Mueller–Hinton broth (Oxoid, Thermo Fisher Scientific, Waltham, MA, USA). The bacterial strain was added to each tube, and the suspensions were adjusted turbidity level of 0.5 on the McFarland scale. The minimal inhibitory concentration (MIC) was expressed as the lowest concentration inhibiting visible growth of bacteria after 24 h of incubation at 37 °C. The positive growth control consisted of *E. coli* in Mueller–Hinton broth without additions, while the negative control for sterility consisted of un-inoculated broth. The experiment was carried out in triplicate, and the cultures were monitored for 48 h.

### 2.4. Calves and Treatments

The randomized uncontrolled in vivo study was conducted on a fattening farm with 650 calves (Romanian spotted × Blanc Blue Belge crosses). The calves are purchased by the farm from multiple sources, consisting of small family farms with 10–15 individuals. They were bought by a house-to-house salesman and taken to the highest bidder. The time between calf purchase and final resale could be anywhere between 6 to 72 h or more. Considering a long time in transit, commingling, and a frequently naïve immune system, according to the farm’s registry, the first signs of disease appeared in the first 48 h at the destination farm (morbidity > 80%). The morbidity was associated with diarrhea, respiratory disease, or a manifestation of both. Modifications such as bloat were seldom identified. Upon arrival, calves were processed and individually weighed in a squeeze chute. Within this farm, sorting was performed in accordance with registered weight: (a) <80 kg calves were housed individually in polypropylene hutches and fed milk replacer for 2 weeks; (b) >80 kg calves were redirected to one of the group pens, with a 20 head capacity, with straw-bedded lying area and concrete feeding alley with 4 individual water feeders. Water and feed are provided ad libitum. Rations consisted of barley straw and 50% corn, 20% barley, 16% soybean meal, 8% sunflower seed meal, 5.7% bran, and 0.3% commercial premix (VitaMiral, Agravis Raiffeisen AG, Münster, Germany). The total protein content was 17%. The majority of the calves were fed straw and were ad libitum suckling in the farm of origin. Therefore, the risk of gorging on an unfamiliar high corn and barley diet was low, as they tended to mainly feed on the familiar straw. All calves entering the farm were pre-weaned and they were considered high-risk calves, with no preconditioning, and no vaccination history.

Eligibility criteria were as follows: calves aged between 60 and 84 days with a mean weight of 101.4 kg were selected after a minimum of 48 h rest and acclimatization after transport, with no behavioral changes (loss of appetite or failure to feed, depression, weakness, dehydration, or bovine respiratory disease symptoms such as unilateral or bilateral ocular and nasal discharge or drooping ears), but diagnosed with diarrhea in the clinical examination.

Formally, the study was conducted according to a two-factor repeated-measure experimental design with 2 fixed effects—the time (9 levels, i.e., day 0 through day 8) and the group (4 levels, i.e., 4 treatment groups), and equal allocation ratio. Since the activated MZC was about to be tested for the first time in vivo, we considered the experiment a phase I clinical trial. Therefore, at the inclusion time, selected calves (*n* = 80) were randomly assigned to 4 equal groups (*n* = 20, sample size).

For better control of the dosage, we suspended the MZC in drinking water (250 mL) and administered individually to each calf. Several doses (from 0.5 g/kg body weight (bw)/day to 1.5 g/kg bw/day) of MZC were evaluated on small groups (4 individuals) for 5 days before the main in vivo testing. On the basis of subsequent clinical observations of the treated calves, we considered the dose of 1 g/kg bw/day of MZC to be the most appropriate for therapeutic use.

For ethical and legal reasons (Directive 2010/63/EU of the European Parliament and the Council and national legislation), we did not include a control group in the experiment. To minimize the bias, we implemented a different therapeutic protocol over a period of 8 days in each of the 4 groups: (1) group A—MZC (1 g/kg bw/day), (2) group B—chestnut extract (20 mg/kg bw/day in drinking water) and oxytetracycline (OXY) (20 mg/kg bw, intramuscular, single dose), (3) group C—chestnut extract (20 mg/kg bw/day) and MZC (1 g/kg bw/day), (4) group D—OXY (20 mg/kg bw, intramuscular, single dose) and MZC (1 g/kg bw/day).

Although OXY could be given orally, for better control of the experiment, we gave the antibiotic intramuscularly. Due to OXY’s pharmacokinetics, the difference in effect on gut microbiota would be minimal, regardless of the route of administration. Considering 50% or more is excreted via bile into the small intestine, effect variation between oral and parenteral routes would be insignificant [17,18].

The in vivo experiment was carried out by 2 blinded veterinary technicians and 1 veterinarian.

### 2.5. Health Status Evaluation

Each calf was examined daily throughout the monitoring period and any alterations of their health status were recorded. Special attention was given to possible adverse effects noticed in the groups that received MZC. Fecal consistency scores and diarrhea severity were assessed daily for each calf, as described by Sadeghi and Shawrang (2008) [19]: 0 = normal (soft without fluid), 1 = soft (semi-solid, mostly solid), 2 = runny (semi-solid, mostly fluid), and 3 = watery (all fluid). Moreover, the body temperature was recorded daily, with rectal temperatures ≥ 39.5 °C considered elevated. One individual from group A showing drooping ears, unilateral eye discharge, and elevated body temperature was removed from the study on day 2 and treated. Slight symptoms such as abdominal cramps and pain, mild depression, and light dehydration were observed during the extent of the study, but were transitory and no other treatments were considered necessary.

All the experiments on animals were carried out in accordance with the international and national legislation and approved by the local commission on bioethical issues (Decision 122/09.11.2018).

### 2.6. Statistical Analysis

The efficacy of anti-diarrhea therapy was assessed using 3 measures: (1) fecal consistency score, which was an ordinal variable including 4 classes denoted by integers from 0 (no diarrhea) through 3 (severe diarrhea); (2) prevalence of diarrhea, which was the percentage of calves with fecal consistency score higher than 0; (3) rate of complete recovery from diarrhea defined as the percentage of calves with complete and sustained disappearance of diarrhea during the observation period. The fecal consistency score was presented as the median and range (or measurements for individual calves in the figure), and for the needs of statistical analysis, we rank-transformed it using type RT-1 transformation [20]. Then, the fecal consistency score was analyzed using the repeated-measure ANOVA. Dunnett’s post hoc test was used to identify the day on which the fecal consistency score decreased significantly when compared to the initial value. The prevalence of diarrhea was presented with a 95% confidence interval (CI 95%) calculated according to the Wilson score method [21] and compared between groups and time points using the Pearson’s chi-squared test. The rate of complete recovery from diarrhea was analyzed using the Kaplan–Meier product-limit estimator with CI 95% calculated with log-minus-log method, and the groups were compared with a log-rank test. All tests were two-sided. A significance level (α) was set at 0.05. Statistical analysis was performed in TIBCO Statistica 13.3.0 (TIBCO Statistics Inc., Palo Alto, CA, USA).

## 3. Results

### 3.1. Cytotoxicity

**Figure 1:**
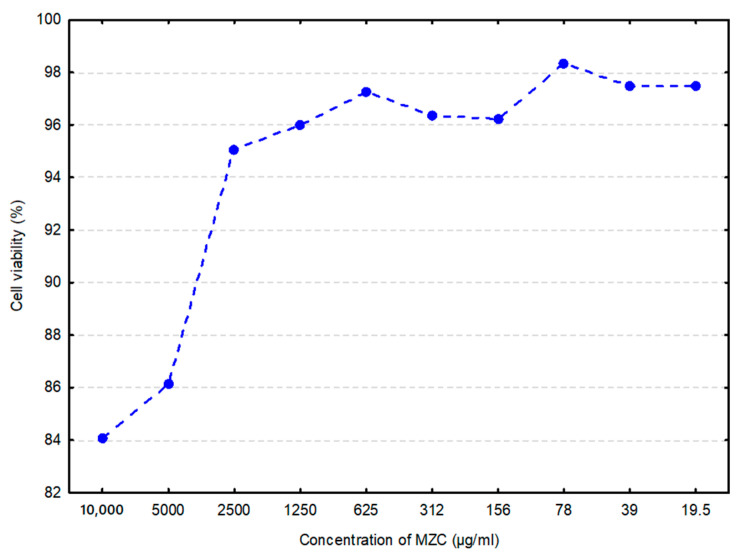
Cell viability of bovine intestinal epithelial cells (BIECs) when treated with zeolite clinoptilolite (MZC) (arithmetic mean of three replications) is shown as a percentage of the negative control. Cells in the culture medium represented the negative control.

Generally, cytotoxicity is considered an essential indicator for toxicity assessment of various molecules or medical devices as it is simple and fast, exhibits high sensitivity, and can prevent animal loss due to poisonous effects [22]. In vitro data (Figure 1) demonstrated ≈16% loss in cell viability by MZC at its highest concentration (10 mg/mL), and less than 3% at its lowest (19.5 μg/mL).

### 3.2. Antimicrobial Activity

The results for MIC showed that the MZC used in our experiment did not explicit a visible antimicrobial effect against *E. coli* at concentrations ranging from 0.156 to 10 mg/mL.

### 3.3. In Vivo Study

**Figure 2:**
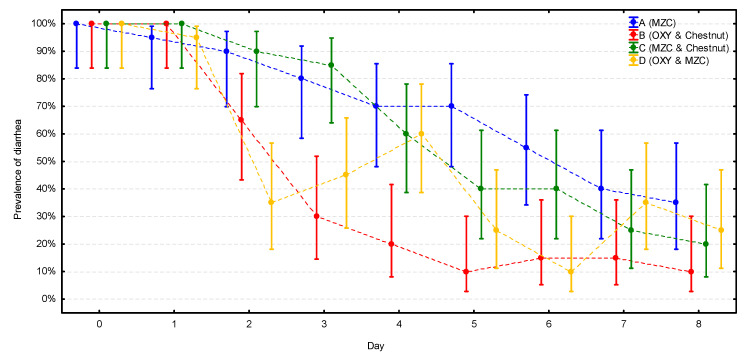
The change of the prevalence of diarrhea (with CI 95%) during the observation period in the four groups (*n* = 20 calves per group). MZC—micronized zeolite clinoptilolite, OXY—oxytetracycline.

At the beginning of the study, all calves in all four groups had diarrhea (Figure 2, Supplementary Materials Table S1). At the end of the study, the prevalence of diarrhea was significantly lower in all four groups (*p* < 0.001), ranging from 10% in group B to 35% in group A, with this difference close to statistical significance (*p* = 0.058). The prevalence followed very similarly, constantly decreasing patterns in two groups without antibiotic treatment (A and C). A stable decrease of prevalence was also observed in group B; however, in this group, the prevalence dropped much quicker and reached the lowest value already on day 5, while in groups A and C, the lowest value was reached at the end (day 8) of the observation. The most unstable course of the enteric disease was observed in group D, where the prevalence dropped first even faster than in group B, reaching 35% on day 2, but increased again to 60% on day 4 (the same level as in groups without the antibiotic); next, it dropped again to 10% on day 6 and increased to 25–35% at the end of the study.

**Figure 3:**
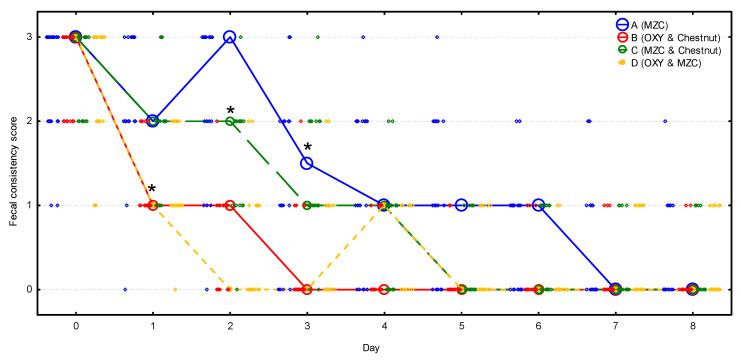
The change of the fecal consistency score (median denoted by circles and individual scores by diamonds) during the observation period in four groups (*n* = 20 calves per group). Asterisks signify the moment when the score decreased significantly compared to the initial value in the group. MZC—micronized zeolite clinoptilolite, OXY—oxytetracycline.

Fecal consistency score was nearly identical in all four groups before the treatment, with a median of 3; nonetheless, during the observation period, it decreased significantly (*p* < 0.001) and differed significantly between groups (*p* < 0.001) (Figure 3, Table S2). It decreased significantly (*p* < 0.001) in groups B and D on day 1, while it was still unchanged in groups A (*p* = 0.999) and C (*p* = 0.102). On day 2, it dropped significantly in group C (*p* = 0.001), whereas it remained unchanged in group A (*p* = 0.870), and then it decreased significantly on day 3 (*p* < 0.001). Median fecal consistency score decreased to 1 in groups B and D on day 1, in group C on day 3, and in group A on day 4. Then, the median fecal consistency score decreased to 0 in group B on day 3, in groups C and D on day 5, and in group A on day 7. All the relapses of diarrhea observed in group D between days 3 and 7 were of mild intensity (score 1).

**Figure 4:**
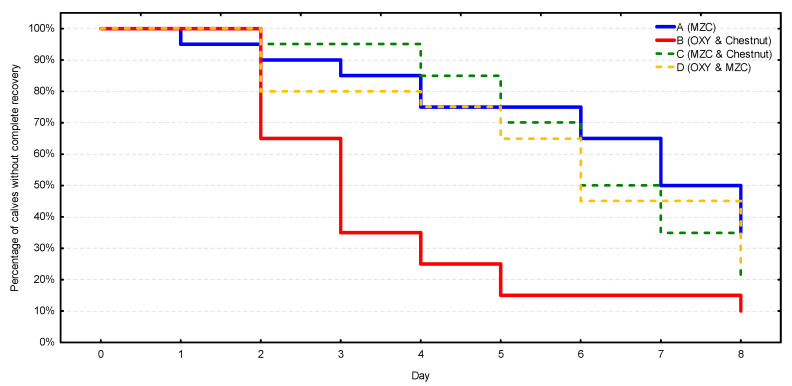
The complete recovery rate from diarrhea (i.e., complete and sustained disappearance of diarrhea) in four groups (*n* = 20 calves/group). MZC—micronized zeolite clinoptilolite, OXY—oxytetracycline.

In terms of the complete recovery (Figure 4, Table S3), the fastest and most stable recovery process was observed in group B, in which 50% of calves got over diarrhea on day 3, 75% on day 4, and 90% on day 8. The remaining three groups were characterized by similar and significantly slower recovery processes (*p* = 0.002) in comparison with group B. Diarrhea disappeared in more than 50% of the calves in these groups as late as between days 6 and 8. Similarly, the recovery rate did not exceed 75% during the observation period.

## 4. Discussion

As previously discussed, the application of zeolites in animal science has long been proposed [23]. Therefore, the results of this study complete the picture of the biomedical application of zeolites by demonstrating the properties of activated MZC in treating diarrhea in fattening calves.

While it has been proven that natural clinoptilolite is well tolerated when tested on phagocytic human cells [24], no studies have been conducted thus far on bovine cells. Following the cytotoxicity assay on BIECs, the MZC proved to be safe for in vivo testing, placing our results in line with data from the literature on zeolites. Furthermore, cell viability tests on BIECs demonstrated a dose-dependent response.

Between-treatment balance was successfully achieved following randomization. The fastest and most constant recovery process with the highest recovery rate was associated with the treatment based on OXY and chestnut. Therefore, the bacterial implication in this study can be suspected on the basis of these results. Nevertheless, the specific etiology has not been established and other causes cannot be ruled out entirely. The aforementioned results are also supported by the well-known effects of antimicrobials in such therapies [8], as well as the well-evidenced efficacy of chestnut tanning against calf diarrhea [11]. Nevertheless, the use of a combination of MZC and chestnut has only a minimal advantage over the exclusive use of MZC, manifested mainly by a slightly faster reduction of diarrhea intensity. As the MZC from the Rupea region had neither bactericidal nor bacteriostatic effects when used directly on microorganisms such as *E. coli*, the effects of clinoptilolite can be explained by its ion exchange and adsorption properties. According to the literature, some zeolites act against a broad spectrum of microorganisms, including Gram-negative and Gram-positive bacteria, but only when they serve as carriers for other molecules [25,26,27,28] or when they are used for their synergic action being combined with antibiotics [13]. While the interaction between inorganic materials and bacterial cells has been reported in numerous publications [29,30,31,32,33,34], it has also been shown that clinoptilolite is able to absorb and partially inactivate enterotoxins of *E.* *coli* [19,35]. It was suggested that the alleviating effect of clinoptilolite on calf diarrhea may result from the alteration of metabolic acidosis through direct effects on osmotic pressure in the gastrointestinal system [19]. In addition, clinoptilolite micronization provided a higher contact surface, while its activation improved the absorption properties [36].

The optimal dosage of MZC used in our study (1mg/kg bw/day) was in agreement with the same dose used by Sadeghi and Shawrang (2008) [19] in a study conducted on newborn Holstein calves. Replacement of chestnut extract with MZC as an adjuvant to OXY was associated with an abrupt disappearance of diarrhea (even faster than when OXY and chestnut were used), followed by relapses of mild diarrhea in roughly one-fourth of calves in the next days.

Therefore, our results open new opportunities regarding the use of MZC for the treatment of this pathology in organic raising, as it has been shown that is little difference between disease occurrence between such systems and conventional dairy cattle farms [37]. Moreover, since human studies have suggested that oral zeolite supplementation is associated with significant immune-modulating effects [38], one important future research direction could be represented by investigations on the prophylactic potential of MZC against diarrhea in calves. If efficient, the activated MZC could represent an important tool in minimizing the antibiotic residues in the food chain or environment.

## 5. Limitations of the Study

Although the etiology of diarrhea was not established, by evaluating data from the farm registers (morbidity at 48 h > 80%) and the results showing that treatment involving antibiotic was the most efficient, we found that bacterial implication, in this case, seemed to be the most probable. Nevertheless, diarrhea due to other factors such as changes in feeding cannot be ruled out.

Due to ethical and legal reasons, another limitation of the study is the absence of a control group and the classification of the in vivo experiment as an uncontrolled clinical trial.

Even though the treatment used did not include fluids and an anti-inflammatory component, the daily clinical examination allowed us to identify just one calf that was eliminated from the experiment and received classical therapy against diarrhea.

Despite the fact that we checked the rectal temperature, we did not score other clinical modifications such as mild abdominal cramps and pain, mild depression, and light dehydration, as the symptoms were transitory and were resolved without further intervention.

## 6. Conclusions

Activated micronized zeolite clinoptilolite represents a safe product that could be used in cattle. Even if the therapy involving antibiotics proved to be superior in this case, MZC could represent a strong alternative that can be used to reduce the amount of antimicrobials currently used for the control of diarrhea in calves, therefore contributing to the reduction of the antimicrobial resistance phenomenon.

## Figures and Tables

**Table 1:** The chemical composition of micronized zeolite clinoptilolite (MZC).

Compound	SiO_2_	Al_2_O_3_	CaO	K_2_O	Fe_2_O_3_	MgO	Na_2_O
Percentage	68.75–71.30%	11.35–13.10%	2.86–5.20%	3.17–3.40%	1.90–2.10%	1.18–1.20%	0.82–1.30%

## Supplementary Materials

The following are available online at https://www.mdpi.com/2076-2615/10/12/2284/s1: Table S1: The change of the prevalence of diarrhea with CI 95% during the observation period in the four groups (*n* = 20 calves/group), Table S2: The change of the fecal consistency score (median and range) during the observation period in the four groups (*n* = 20 calves/group), Table S3: The complete recovery rate from diarrhea (i.e., complete and sustained disappearance of diarrhea) presented as the Kaplan–Meier estimator with log-log CI 95% in the four groups (*n* = 20 calves/group).
