# Supplementary material for: The Use of Activated Micronized Zeolite Clinoptilolite as a Possible Alternative to Antibiotics and Chestnut Extract for the Control of Undifferentiated Calf Diarrhea: An In Vitro and In Vivo Study
Source: Animals (Basel). 2020 Dec 3;10(12):2284. doi: 10.3390/ani10122284 (PMC7761744; doi:10.3390/ani10122284)
Supplement: Supplementary file 1 [file animals-10-02284-s001.pdf]

Supplementary file

Table S1. The change of the prevalence of diarrhea with CI 95% during the observation period in the 4 groups (n=20 calves/group).

Group	Day								
	0	1	2	3	4	5	6	7	8
A (MZC)	100 (83.9, 100)	95 (76.4, 99.1)	90 (69.9, 97.2)	80 (58.4, 91.9)	70 (48.1, 85.5)	70 (48.1, 85.5)	55 (34.2, 74.2)	40 (21.9, 61.3)	35 (18.1, 56.7)
B (OXY & Chestnut)	100 (83.9, 100)	100 (83.9, 100)	65 (43.3, 81.9)	30 (14.5, 51.9)	20 (8.1, 41.6)	10 (2.8, 30.1)	15 (5.2, 36)	15 (5.2, 36)	10 (2.8, 30.1)
C (MZC & Chestnut)	100 (83.9, 100)	100 (83.9, 100)	90 (69.9, 97.2)	85 (64, 94.8)	60 (38.7, 78.1)	40 (21.9, 61.3)	40 (21.9, 61.3)	25 (11.2, 46.9)	20 (8.1, 41.6)
D (OXY & MZC)	100 (83.9, 100)	95 (76.4, 99.1)	35 (18.1, 56.7)	45 (25.8, 65.8)	60 (38.7, 78.1)	25 (11.2, 46.9)	10 (2.8, 30.1)	35 (18.1, 56.7)	25 (11.2, 46.9)

Table S2. The change of the fecal consistency score (median and range) during the observation period in 4 groups (n=20 calves/group).

Group	Day								
	0	1	2	3	4	5	6	7	8
A (MZC)	3 (1-3)	2 (0-3)	3 (0-3)	1.5 (0-3)	1 (0-3)	1 (0-3)	1 (0-2)	0 (0-2)	0 (0-2)
B (OXY & Chestnut)	3 (2-3)	1 (1-2)	1 (0-2)	0 (0-2)	0 (0-1)	0 (0-1)	0 (0-1)	0 (0-1)	0 (0-1)
C (MZC & Chestnut)	3 (2-3)	2 (1-3)	2 (0-3)	1 (0-3)	1 (0-2)	0 (0-1)	0 (0-1)	0 (0-1)	0 (0-1)
D (OXY & MZC)	3 (1-3)	1 (0-2)	0 (0-2)	0 (0-2)	1 (0-1)	0 (0-1)	0 (0-1)	0 (0-1)	0 (0-1)

Table S3. The complete recovery rate from diarrhea (i.e. complete and sustained disappearance of diarrhea) presented as the Kaplan-Meier estimator with log-log CI 95% in 4 groups (n=20 calves/group)

Group	Day								
	0	1	2	3	4	5	6	7	8
A (MZC)	100	95 (69.5, 99.3)	90 (65.6, 97.4)	85 (60.4, 94.9)	75 (50, 88.7)	75 (50, 88.7)	65 (40.3, 81.5)	50 (27.1, 69.2)	35 (15.7, 55.2)
B (OXY & Chestnut)	100	100	65 (40.3, 81.5)	35 (15.7, 55.2)	25 (9.1, 44.9)	15 (3.7, 33.5)	15 (3.7, 33.5)	15 (3.7, 33.5)	10 (1.7, 27.2)
C (MZC & Chestnut)	100	100	95 (69.5, 99.3)	95 (69.5, 99.3)	85 (60.4, 94.9)	70 (45.1, 85.3)	50 (27.1, 69.2)	35 (15.7, 55.2)	20 (6.2, 39.3)
D (OXY & MZC)	100	100	80 (55.1, 92)	80 (55.1, 92.0)	75 (50, 88.7)	65 (40.3, 81.5)	45 (23.1, 64.7)	45 (23.1, 64.7)	25 (9.1, 44.9)
